# Neuroradiological patterns and prognostic implications in type I Alexander disease

**DOI:** 10.1016/j.ymgme.2026.110189

**Published:** 2026-06-15

**Authors:** Ylenia Vaia, Filippo Arrigoni, Liat Ben Sira, Geneviève Bernard, Enrico Bertini, Odile Boespflug-Tanguy, Fabio Bruschi, Cristina Cereda, Florian Eichler, Alessandra Erbetta, Simona Ferraro, Morteza Heidari, Luca Lalli, Gabrielle Lambert, Stephanie Libzon, Daniela Longo, Isabella Moroni, Amanda Nagy, Francesco Nicita, Florence Renaldo, Diana Rodriguez, Golazin Shahbodagh Khan, Ali Reza Tavasoli, Ellen Winter, Ayelet Zerem, Cecilia Parazzini, Davide Tonduti

**Affiliations:** a C.O.A.L.A. (Center for Diagnosis and Treatment of Leukodystrophies), Unit of Pediatric Neurology, V. Buzzi Children’s Hospital, Milan, Italy; b Neuroscience Research Center, Department of Biomedical and Clinical Sciences, University of Milan, Milan, Italy; c Unit of Pediatric Radiology and Neuroradiology, V. Buzzi Children’s Hospital, Milan, Italy; d Pediatric Radiology, Department of Radiology, Tel Aviv Sourasky Medical Center; Gray Faculty of Medical and Health Sciences, Tel Aviv University, Tel Aviv, Israel; e Departments of Neurology and Neurosurgery, Pediatrics, and Human Genetics, McGill University, Montreal, Quebec, Canada; f Department of Specialized Medicine, Division of Medical Genetics, McGill University Health Centre, Montreal, Quebec, Canada; g Child Health and Human Development Program, Research Institute of the McGill University Health Centre, Montreal, Canada; h Research Unit of Muscular and Neurodegenerative Disorders, IRCCS Bambino Gesù Children’s Hospital, Rome, Italy; i AP-HP, Centre de reference maladies rares « LEUKOFRANCE », Neurologie Pédiatrique, Université Paris-Cité, INSERM-UMR1141, Hôpital Robert-Debré, Paris, France; j Center of Functional Genomics and Rare Diseases, Dept. of Pediatrics Buzzi Children’s Hospital, Milan, Italy; k Pediatric Department, Buzzi Children’s Hospital, Milan, Italy; l Harvard Medical School, Boston, MA, USA; m Department of Neurology, Massachusetts General Hospital, Boston, MA, USA.; n Neuroradiology Unit, Fondazione IRCCS Istituto Neurologico Carlo Besta, Milan, Italy; o Pediatric Neurology Division, Department of Pediatrics, Children’s Medical Center, Pediatric Center of Excellence, Tehran University of Medical Sciences, Tehran, Iran; p Pediatric Neurology Division, Myelin Disorders Clinic, Children’s Medical Center, Pediatric Center of Excellence, Tehran University of Medical Sciences, Tehran, Iran.; q Unit of Translational Immunology, IRCCS Foundation National Cancer Institute, Milan, Italy; r Pediatric Neurology Institute, Dana-Dwek Children’s Hospital, Tel Aviv Sourasky Medical Center, Tel Aviv, Israel; s Sackler Faulty of Medicine, Tel Aviv University, Tel Aviv, Israel; t Neuroradiology Unit, Department of Imaging, IRCCS Bambino Gesù Children’s Hospital, Scientific Institute for Research and Health Care, Rome, Italy; u Department of Pediatric Neurosciences, Fondazione IRCCS Istituto Neurologico Carlo Besta, Milan, Italy; v Unit of Muscular and Neurodegenerative Diseases, IRCCS Bambino Gesù Children’s Hospital, Rome, Italy; w Department of Pediatric Neurology, Reference Center for Rare Neurogenetic Diseases, Armand-Trousseau Hospital, APHP, Sorbonne University, Paris, France; x Department of Neurology, Barrow Neurological Institute, Phoenix Children’s Hospital, Phoenix, AZ, USA

## Abstract

**Introduction:**

Type I Alexander Disease (AxD) is a rare leukodystrophy that can be subclassified into subtypes (Ia, Ib, Ic, and Id), in order of decreasing severity. To date, no study has correlated MRI findings with AxD type I clinical evolution. This study aims to identify MRI features that may predict disease progression.

**Methods:**

Patients with genetically confirmed type I AxD were recruited from multiple Leukodystrophy Centers worldwide. Clinical data were collected via RedCap, and patients were retrospectively classified by disease subtype. Leukodystrophy experts analyzed the first available MRI scans using an adapted AxD MRI scoring protocol, and imaging findings were correlated with disease subtypes.

**Results:**

47 subjects with type I AxD were enrolled. Subtype distribution included 3 Ia, 12 Ib, 8 Ic, 10 Id, 10 Ic/Id, and 4 undetermined. Risk of belonging to type Ib compared to other subtypes was significantly increased in case of involvement of parietal subcortical WM (OR = 16.1, *p* = 0.003), occipital deep WM (*p* = 0.008), temporal deep (*p* = 0.018), and periventricular WM (*p* = 0.013), body of corpus callosum (*p* = 0.018), genu of corpus callosum (*p* = 0.047) and hilar region of cerebellum (*p* = 0.008). Involvement of occipital periventricular WM was associated to a higher risk of being type Ic compared to type Id (*p* = 0.010).

**Conclusions:**

Some distinct MRI patterns on initial MRI scans may predict disease outcomes in type I AxD. These findings may help clinicians in early prognostic stratification, with key brain regions needing careful evaluation. Further studies are required to validate these findings and improve prognostic accuracy in type I AxD.

## 1. Introduction

Alexander disease (AxD) is a rare, progressive leukodystrophy resulting from autosomal dominant gain-of-function pathogenic variants in the Glial Fibrillary Acidic Protein (*GFAP*) gene, which encodes the primary intermediate filament protein in astrocytes [1]. Clinically, AxD presents with a wide range of phenotypes, and multiple disease classifications are currently in use [2–5]. Prust et al. identified two types of AxD based on clinical and neuroradiological features at onset: type I, characterized by early onset, classic neuroradiologic features, seizures, macrocephaly, and developmental delay, and type II, which can manifest at any age and is characterized by atypical radiologic features, primarily bulbar symptoms, and autonomic dysfunction [2]. Recently, a subclassification of type I AxD patients has been proposed, which also takes into account the clinical evolution over time [6]. In particular, given the extreme clinical heterogeneity, type I AxD patients may be subclassified in a type Ia, characterized by absence of postural acquisition and early fatal course; type I b, characterized by some postural acquisition but no autonomous ambulation, and usually worsening before the 6th year of life; type Ic, characterized by acquisition of autonomous ambulation with delay, followed by deterioration before the onset of adolescence (i.e., 12 years) and type Id, characterized by clinical stability beyond the onset of adolescence [6]. While defining some shared disease trajectories that may be useful for patients’ counseling as well as enrolling and monitoring in a Clinical Trial context, this model entails the challenge of being only applicable in a retrospective fashion, and no disease biomarkers are currently able to anticipate disease evolution [7,8], and thus belonging to specific disease subtypes, from the early stages of disease. No disease-modifying treatment is currently available for AxD, and management is mainly supportive [9]. Recent advances in diagnostics and drug development have led to the emergence of therapeutic candidates for clinical trials [10], making the selection of suitable biomarkers for disease progression and the definition of appropriate endpoints crucial.

Brain Magnetic Resonance Imaging (MRI) pattern recognition is crucial for guiding the diagnostic process in AxD. In fact, prior to identification of the causative genetic etiology for AxD, van der Knaap et al. defined five MRI criteria for the neuroimaging-based diagnosis of the disease [11]. However, to date, no studies have explored the correlation between early MRI findings and disease clinical characteristics and progression.

We aimed to investigate specific MRI features that may predict clinical disease trajectory over time and based on that, to create a prognostic MRI scoring system, intended to stratify patient based on MRI severity. Moreover, we also aimed to create a prognostic flowchart combining clinical and MRI findings.

## 2. Methods

### 2.1. Setting and participants

Patients were identified from several Leukodystrophy Centers worldwide (V. Buzzi Children’s Hospital, Milan; Armand-Trousseau Hospital, Paris; Children’s Medical Center, Tehran; Dana-Dwek Children’s Hospital, Tel Aviv Sourasky Medical Center, Tel Aviv; IRCCS Istituto Neurologico Carlo Besta, Milan; IRCCS Bambino Gesù Children’s Hospital, Rome; Massachusetts General Hospital, Boston; McGill University Health Centre, Montreal, Canada). Inclusion criteria were molecular confirmation of AxD and classification based on Prust et al. as Type I AxD, as per clinical picture and neuroimaging findings [2].

Relevant clinical and neuroradiological data including *GFAP* pathogenic variants, age at disease onset, age at first MRI, developmental milestones (acquisition of head control, trunk control, ambulation with aid, independent ambulation) and disease progression (loss of previously acquired developmental milestones) were retrospectively abstracted from the medical records at each institution and collected via RedCap© tool hosted at V. Buzzi Children’s Hospital in the form of RedCap© [12,13] surveys distributed to each participating institution.

Phenotypic subgroup assignment was based on the clinical criteria derived from previously published phenotype descriptions [6,14]. The data necessary for subclassification were requested from each enrolling clinician, and assignment to each category was performed by the first author based on the available clinical information. Cases for which sufficient clinical data were not available were not assigned to a subtype and therefore not included in the study.

### 2.2. Materials

An MRI evaluation protocol was developed specifically for AxD by adapting existing MRI scoring systems used for other leukodystrophies, including X-ALD, POLR3 and PMD (see Supplementary File) [15–17]. The adapted protocol was tailored to capture the distinctive neuroradiological features of AxD. It consisted in four sections: (1) white matter (WM) signal abnormalities assessed across supratentorial and infratentorial regions (2) “mass-like” lesions; (3) contrast enhancement (CE); and (4) atrophy scoring. For the purpose of the present analyses, only the variables derived from the first section, in particular the presence, absence, and localization of WM signal abnormalities, were included. The remaining sections were collected as exploratory observations and will be the subject of future analyses. The available MRI scans were evaluated by two pediatric neuroradiologists (F.A. and C.P., each with over 10 years of experience in pediatric neuroradiology) blinded to patients’ clinical data, during online or in-person consensus meetings attended by the clinical team from the coordinating institution (Y.V. and D.T.), as well as the clinicians and radiologists from the respective site.

Only information regarding the first available MRI for each patient was included in the statistical analysis to evaluate the prognostic value of the neuroradiological findings at first evaluation. For the same reason, patients with a time to first available MRI >4 years were excluded from the analyses. No follow up MRIs were included in the study.

For patients belonging to the type Ia group, due to the very early age at first available MRI and the resulting physiological lack of myelin, the score was not applied, and only a consensus qualitative evaluation among the neuroradiologists was performed.

### 2.3. Statistical analysis

Categorical variables were reported as percentages and continuous variables as median and interquartile range (IQR) according to the distribution. Differences in clinical variables were tested using the Mann Whitney and Kruskal Wallis tests for continuous data and the Fisher test for categorical data.

Univariate logistic regression models were applied to explore associations between clinical variables and outcomes, while a multinomial logistic regression model was used to evaluate predictors across multiple clinical outcomes. A two-sided significance level of 5% was adopted for all analyses. Statistical analyses were performed using R software (version 4.4.1, R Foundation for Statistical Computing, Vienna, Austria).

### 2.4. MRI scoring system

After identifying MRI areas significantly associated with an increased risk of belonging to a more severe disease phenotype (Ib), we aimed to develop a prognostic MRI scoring system. The goal of this scoring system was to identify individuals at a higher risk of not acquiring independent ambulation.

Areas shown to have predictive value in statistical analyses comparing Ib to other subtypes with a *p* < 0.02 were selected for inclusion in the scoring system. Each patient was assigned a score of 1 based on the involvement of each of these selected areas (affected area = 1 vs non-affected area = 0), for a total of 5/5 if all areas were involved. The scores were then grouped into three categories based on severity: <2, 2–3, and >3. Finally, a logistic regression model was applied to assess the association between these score categories and the disease outcomes, intended as disease subtypes (Ib vs others), using the 2–3 group as the reference category.

### 2.5. Flowchart for early prediction of phenotype

A flowchart was created to facilitate the early-stage identification of AxD subtypes by integrating MRI scoring criteria with clinical data. The thresholds and criteria were determined based on existing literature, clinical observations, and prior analyses. Clinical data focused on the acquisition of two key developmental milestones, such as head control and autonomous ambulation, as key discriminators emerging from the subclassification based on disease evolution over time [6].

Head control was assessed as the age at which the patient achieved stable head control, with a threshold set at the 99th percentile of head control acquisition calculated from data abstracted from both our cohort and a cohort previously described in the literature, for which data regarding age at developmental milestones acquisition was available [6]. Similarly, autonomous ambulation was defined as the ability to walk independently, with a threshold also set at the 99th percentile of acquisition of autonomous ambulation in the same two cohorts. The flowchart combined developmental milestones and MRI scores into a stepwise decision-making framework.

## 3. Results

### 3.1. Demographics

A total of 47 subjects affected by genetically confirmed type I AxD were enrolled. 3 subjects were classified as presenting as type Ia AxD, 12 as Ib, 8 as Ic, 10 as Id, 10 as Ic/Id and 4 were unclassifiable due to too young age or lack of comprehensive information. 3 patients (1 Ic, 1 Id, 1 Ic/Id) were excluded from the quantitative analysis due to time to first MRI available >4 years. A total of 37 patients was then considered for analysis. Median age at disease onset was 1.2 y (Q1–Q3 0.5–1.5). Median age at first MRI was 2.6 y (Q1–Q3 1.8–4.1). Median time from disease onset to first available MRI was 1.7 y (Q1–Q3 0.8–2.5).

### 3.2. Neuroradiological patterns in subtypes Ia

Qualitative analysis of type Ia patients’ brain MRI showed diffuse involvement of supratentorial WM. An antero-posterior gradient remained recognizable, although delineating the precise boundary between normal and pathologically unmyelinated WM was challenging given the young age of the patients. Basal ganglia appeared massively affected, as well as brainstem and particularly the medulla oblongata. Cerebellar WM and the hilar region of the cerebellum appeared transversely involved, and there was global cerebral swelling. Obstructive hydrocephalus was observed in one out of the three patients.

The combination of the severe clinical manifestation and distinctive neuroradiological features enabled the classification of these cases as type Ia d from the early stages of the disease.

### 3.3. Neuroradiological patterns in subtypes other than Ia

Overall, WM was mostly affected in the frontal lobes, with 100% involvement across all subtypes, especially in periventricular and deep regions, while subcortical WM was more rarely involved overall (Table 1).

Basal ganglia involvement was common across all subtypes, with type Ib showing the highest frequency, while thalamic abnormalities were less frequent, observed in about one-third of cases. In the posterior fossa, the cerebellar hilus and WM were most affected in type Ib, with lower rates in types Ic and Id. Brainstem involvement varied: the mesencephalon and pons showed moderately frequent involvement, especially in type Ib, while the medulla oblongata was almost universally affected across all subtypes (over 85%), consistently showing the presence of the characteristic *“Chipmunk sign”* [18]. The spectrum of WM abnormalities across different anatomical regions is shown in Fig. 1.

All subtypes exhibited varying degrees of atrophy at first MRI, with scores ranging from 0/8 to 7/8. In the Ib subtype, the scores were relatively evenly spread, with the largest groups falling at scores of 2 (3/12, 25%) and 3 (3/12, 25%), indicating a moderate range of atrophy in this group. The Ic subtype showed a higher percentage of patients with a score of 1 (43%). One patient in the Ic group had a score of 4, indicating a more pronounced atrophy. Of note, the first available MRI for this patient was at the age of 4.9 years, with a time to first available MRI of 3.4 years. The Id subtype had the highest percentage of patients scoring 2 (33%), followed by scores of 1 (22%) and 0 (22%), reflecting a tendency toward milder atrophy. Only one patient exhibited a score of 4, with his MRI having been performed at 4.1 years of age (2.6 years from disease onset).

A unilateral “mass-like lesion” at the level of the medulla oblongata was observed in 1 Ib, 1 Ic, and 1 Id patients. 1 Ic/Id patient presented with a bilateral “mass-like lesion” at the same level. Additionally, a Id patient exhibited a “mass-like lesion” at the level of the mesencephalon. In addition, “swelling” of limbic and/or diencephalic structures, such as optic chiasm, hypothalamus, fornices, hippocampi, or amygdala, was observed in 45.94% (17/37) of subjects. Specifically, the optic chiasms were involved in 27% (10/37), the hypothalamus in 19% (7/37), and the fornices in 21.62% (8/37) of subjects. Only one subject showed swelling of the hippocampi, and one other of the amygdala bilaterally. Post-contrast images were available in 70.27% (26/37) of cases. Contrast enhancement was present in 75% (6/8) of type Ib, 57.1% (4/7) of type Ic, 50% (3/6) of type Ic/Id, and 40% (2/5) of type Id.

### 3.4. Univariate regression analyses for predicting AxD subtype classification

#### 3.4.1. Ib vs others

Logistic regression showed higher risk of being classified as type Ib compared to all other subtypes in case of involvement of parietal subcortical WM (OR = 16.1 (95% CI: 2.9–133.7), *p* = 0.0032), occipital deep WM (OR = 19.6 (95% CI: 3.1–388.4) *p* = 0.0082), deep (OR = 14.0 (95% CI: 2.2–276.4) *p* = 0.0184) and periventricular temporal WM (OR = 8.9 (95% CI: 1.8–66.8) *p* = 0.013), body of corpus callosum (OR = 7.3 (95% CI: 1.5–44.1) *p* = 0.0182) and hilar region of cerebellum (OR = 10.6 (95%CI: 2.2–80.7) *p* = 0.0076), corpus callosum genu (OR = 4.4 (95% CI: 1.0–20.7) *p* = 0.047) (Fig. 2).

#### 3.4.2. Ic vs Id

When comparing subtypes Ic vs Id, a higher risk of being type Ic was observed in case of involvement of occipital periventricular WM (OR = 48, (95% CI: 3.7–1951) *p* = 0.011).

### 3.5. MRI scoring system development

The areas included in the score were parietal subcortical WM, hilar region of cerebellum, occipital deep WM, and temporal deep and periventricular WM. The subjects were given a score of 0 or 1 based on the involvement of each area, with a minimum score of 0/5 (less affected) and a maximum of 5/5 (all areas involved), with the distribution as follows: 7 patients scored 0, 8 scored 1, 5 scored 2, 7 scored 3, 1 scored 4, 9 scored 5. The relationship between the score and final outcomes was subsequently analyzed, with two outcome categories identified: “Others” (Ic + Ic/Id+Id) and Ib.

Analysis of scores by outcome showed that scores of 0 and 1 were never associated with Ib outcome. In contrast, the highest score (5/5) was more frequently associated with the “Ib” outcome.

The scores were then grouped into three categories (<2 (*n* = 15), 2–3 (*n* = 12), and >3 (10)).

Logistic regression analysis revealed that scores >3 were associated with a significantly higher risk of “Ib” outcome with an OR of 8 (95% CI: 1.28–73.05; *p* = 0.038), indicating a strong relationship between higher scores, and thus the involvement of more than 4 of the selected areas, and the disease severity.

### 3.6. Flowchart for early prediction of phenotype

The MRI scoring system was combined with clinical data such as the acquisition of head control and autonomous ambulation, which are key milestones in the type I AxD subclassification model, to create a flowchart aimed at identifying the likely AxD subtype from the early stages of the disease (Fig. 3).

### 3.7. Definition of thresholds

Data regarding age at acquisition of head control and autonomous ambulation in our cohort (*N* = 41) and a cohort described in literature (*N* = 46) were analyzed to define the thresholds for the flowchart. Head control acquisition was observed in 38/41 patients from our cohort and 29/46 from the literature cohort (77% of the total cohort). Among these, precise age at head control was available for 20 patients from our cohort and 17 patients from the literature (*N* = 37; 42% of the cohort). No outliers were identified. Mean age at head control was: 4 months (SD 2 months). 99° percentile resulted 9 months, that was considered as the threshold value. Autonomous ambulation was acquired in 27/41 patients from our cohort and 14/46 patients from the literature cohort (47% of the total cohort). Age at autonomous ambulation was available for all patients from our cohort and 9 patients from the literature (*N* = 36; 41,37% of the total cohort). Two outliers were identified using the formula μ − SD, μ + kSD (μ = mean, k = parameter of sensitivity and SD = standard deviation), 5 years and 7.4 years, that were excluded. Mean age at autonomous ambulation was: 1.98 years, SD 0.65 years. 99° percentile resulted 3.5 years, that was considered as the threshold value.

Thresholds for MRI scoring system were abstracted from the previous analysis.

### 3.8. Flowchart

Cases were initially stratified by MRI scores into three severity categories: <2, 2–3, and >3.

For MRI scores <2, per our previous observations, the presence of occipital periventricular WM involvement needs to be assessed: patients with occipital periventricular WM involvement are more likely to develop as type Ic, while those without involvement of this area may be more possibly presenting as type Id.

For MRI scores of 2–3, the acquisition of independent ambulation before 3.5 years of age serves as the main criterion. Cases achieving independent ambulation <3.5 years are further stratified based on occipital periventricular WM involvement to differentiate between types Ic and Id. Conversely, cases that don’t acquire independent ambulation are assessed for the acquisition of head control before 9 months of age, with head control <9 months expectedly indicating type Ib while head control >9 months more likely indicating a type Ia. Finally, for MRI scores >3, the acquisition of head control before 9 months is the primary criterion; cases achieving head control <9 months are categorized as type Ib, while those failing to achieve this milestone are likely classified as type Ia.

This framework combines MRI findings and developmental milestones to support the prospective classification of type I AxD into distinct subtypes.

## 4. Discussion

Alexander disease is a rare leukodystrophy with highly heterogeneous clinical presentations, making prognostic assessment particularly challenging. Previous studies have highlighted that the acquisition of developmental milestones such as head control and autonomous ambulation are prognostically significant clinical features [6,14]. In this study, we established threshold ages beyond which the likelihood of achieving these milestones decreases.

In addition, we analyzed the neuroradiological patterns of a large cohort of type I AxD patients. MRI is a proven fundamental part of the diagnostic work up in patients affected by AxD [11], and in our cohort, it proved to be a reliable tool in reflecting the clinical evolution of the disease.

Overall, the frontal WM was consistently affected across all disease subtypes, while the parietal, occipital, and temporal WM showed progressively less frequent involvement, confirming the anteroposterior gradient already described in AxD [11]. The basal ganglia and brainstem were also consistently affected across subtypes. These findings align with the well-known pattern of abnormalities typically seen in type I AxD, fulfilling the classic neuroradiological criteria defined by van der Knaap et al [11].

The goal of this study was to determine whether specific MRI features could predict disease progression from early stages. Type Ia AxD patients typically present with an earlier onset and more severe symptoms, with lack of postural acquisitions and early death, making them easier to identify from early disease stages. The focus of this study was therefore to identify features capable of predicting outcomes toward type Ib, Ic, or Id. We observed that abnormalities tended to involve the deep WM most frequently, followed by the periventricular WM, and, lastly, by the subcortical WM. This pattern of distribution should be interpreted alongside the observation that the anterior areas are more commonly affected than the posterior regions. Consequently, it appears reasonable that the occipital periventricular area is more sensitive to changes in less severe phenotypes. This was also suggested by our analyses, where this area helped differentiate between types Ic and Id. These findings may assist clinicians identifying patients at higher risk of losing autonomous ambulation before the onset of adolescence (Ic), highlighting the importance of closely examining this area on MRI for prognostic stratification in ambulatory AxD patients.

The involvement of parietal subcortical, occipital deep, temporal periventricular and deep WM, as well as body and genu of corpus callosum and hilar region of cerebellum was associated to a higher risk of being a type Ib AxD rather than Ic or Id, thus being associated with a higher risk of never acquiring autonomous ambulation in our cohort. This second set of observations was summarized into a prognostic MRI scoring system to differentiate type Ib AxD from types Ic and Id. Existing MRI scoring systems in leukodystrophies are typically conceived as severity scores used for disease monitoring [16,17,19,20]. Unlike severity scores, a prognostic score is designed specifically for the initial phases of the disease. Therefore, atrophy was not included in our score, being better suited to indicate disease progression. It should be noted that atrophy did not demonstrate any prognostic value in our cohort. This may be attributed to its intrinsic dependence on the time passed between disease onset and MRI, as it progressively increases with disease progression.

The MRI evaluating system demonstrated its reliability as a tool for assessing the risk of belonging to a more severe AxD subtype (type Ib), making it a valuable resource for prognostic stratification during the early disease stages. However, the relatively narrow score ranges used for severity stratification (<2, 2–3, and >3) may be subject to overlap, potentially limiting discrimination between categories. The proposed thresholds should therefore be considered exploratory stratification tools within this pilot cohort rather than definitive severity cutoffs, and larger prospective studies are needed to refine these thresholds and determine their predictive utility. One of the strengths of the proposed scoring system is its applicability to standard MRI protocols. Score calculation only requires axial T2-weighted and/or FLAIR sequences for assessment of lesion burden, at least one axial T1-weighted sequence to evaluate possible cystic changes, and a sagittal sequence of any weighting for evaluation of the brainstem and corpus callosum.

Moreover, this study provides a structured flowchart that may be valuable when addressing clinical prognostic evaluations or disease stratification in the context of clinical trials in AxD.

Although identifying definitive markers to differentiate disease subtypes remains challenging, our data indicate involvement of specific brain regions on MRI and attainment of specific developmental milestones may be helpful in stratifying.

This study has several limitations. Being it a retrospective study, data was sometimes incomplete, and it was not always possible to subclassify patients due to their young age or too short follow-up, leading to an effective small sample size. Moreover, in a few cases time from disease onset to first available MRI was relatively long, thus jeopardizing the effective prognostic value of the assessment. Lastly, MRI scans were reviewed jointly by two pediatric neuroradiologists in consensus rather than through independent blinded scoring, and therefore formal inter-rater reliability analysis was not performed. Therefore, the proposed MRI scoring system should be considered preliminary and non-validated, as it was derived from a relatively small retrospective cohort; future validation studies in independent, prospective populations are needed to assess inter-rater agreement and establish the reproducibility, clinical applicability, and prognostic utility of the scoring system.

In conclusion, these findings underscore the importance of precise regional assessment in neuroimaging studies to better understand the phenotype differentiation in AxD, highlighting some key areas that need to be considered in the formulation of prognostic hypotheses in patients with type I AxD. Moreover, this study provides a structured flowchart that may be valuable when addressing clinical prognostic evaluations or disease stratification in the context of clinical trials in AxD.

PVWM = Periventricular White Matter; SCWM = Subcortical White Matter; AA = Autonomous Ambulation; HC = Head Control.

## Supplementary Material

Supplementary material 1. MRI evaluation protocol

Supplementary material 2. MRI evaluation protocol

## Figures and Tables

**Fig. 1.**
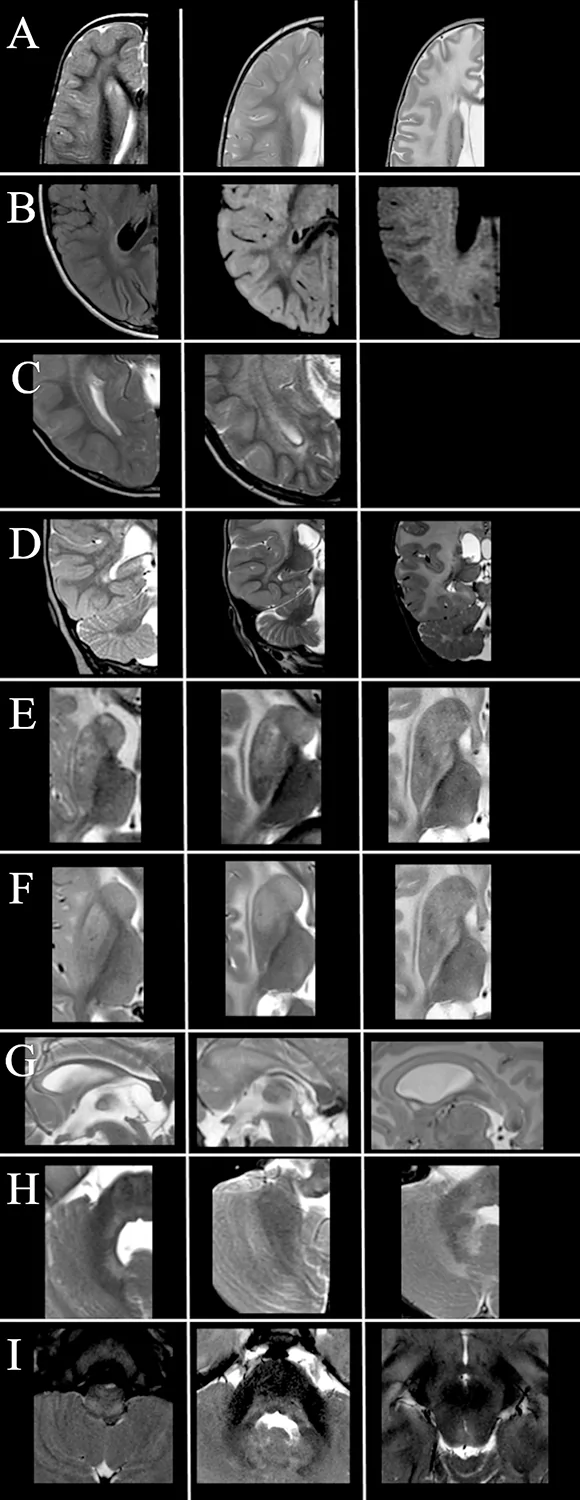
Spectrum of brain MRI abnormalities in Alexander disease across different anatomical regions. WM abnormalities were assessed on T2-weighted images. Hemispheric WM involvement was defined as “bilateral T2-hyperintensity” when more than 50% of the WM in the specific area was involved, bilaterally. The figure illustrates the varying patterns and degrees of lesion involvement observed in Type I AxD, organized by brain region (A–I), with representative images for each pattern. (A) Frontal white matter: involvement limited to deep WM; both periventricular and deep WM; or diffuse involvement of all frontal WM. (B) Parietal white matter: periventricular involvement; deep WM involvement; or diffuse WM involvement. (C) Occipital white matter: periventricular; or combined periventricular and deep WM involvement. (D) Temporal white matter: periventricular; periventricular and deep; or diffuse involvement of all temporal WM. (E) Deep gray nuclei: involvement of putamen and caudate with spared globus pallidus and thalamus; involvement of putamen, caudate, and pallidus with spared thalamus; or all deep gray nuclei affected. (F) Internal capsule: normal appearance; involvement of anterior limb (ALIC) with spared posterior limb (PLIC); or involvement of both ALIC and PLIC. (G) Corpus callosum: lesions restricted to the genu; involving both genu and body; or diffuse involvement of the entire corpus callosum. (H) Cerebellum: selective involvement of hilar region of cerebellum; only deep cerebellar WM involvement; or combined hilar region and deep WM of cerebellum involvement. (I) Brainstem: lesions located in the medulla, pons, or mesencephalon.

**Fig. 2.**
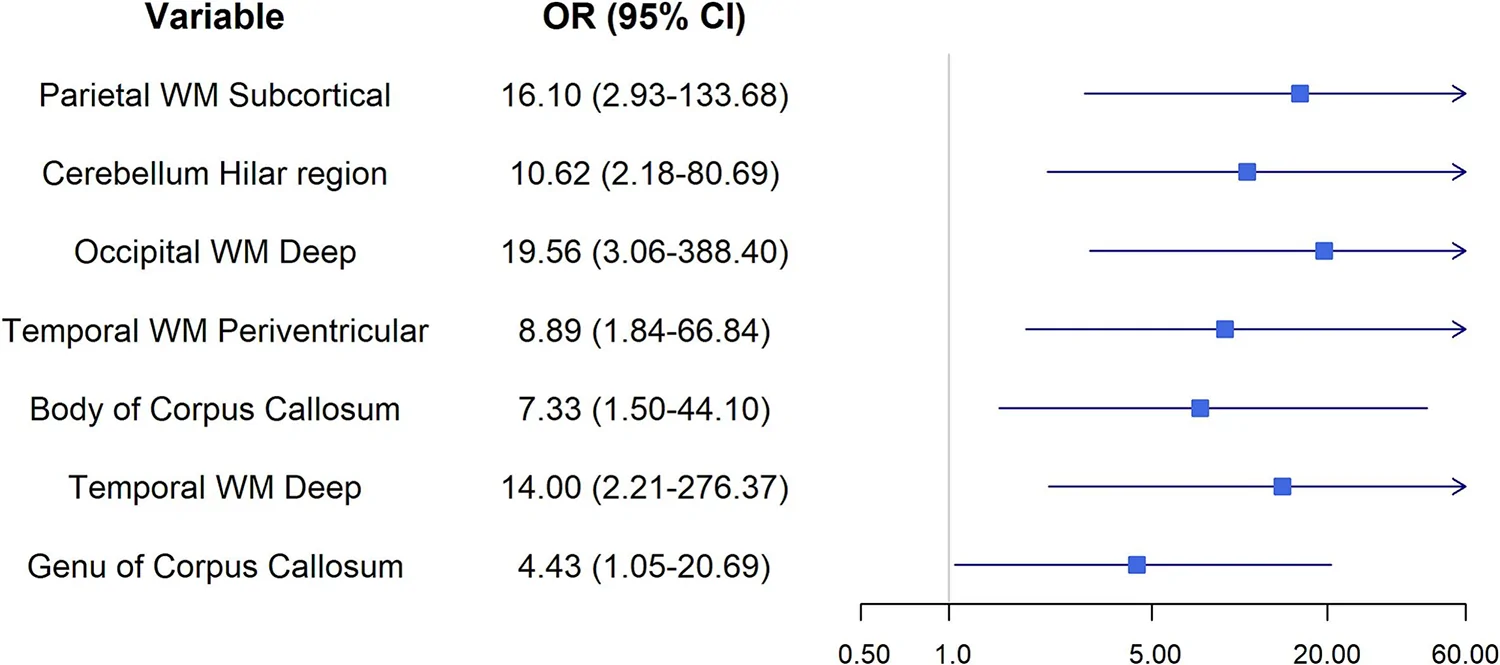
Forest plot of the odds ratio (OR) obtained from the univariate logistic regression with outcome Ib vs others. OR estimates (bold square) and confidence intervals (CI, horizontal lines) describe the association between the 7 involved areas and outcome (Ib subtype). Involved areas identified as risk factors are characterized by both estimates and CI falling on the right. The risk increases as the square moves to the right.

**Fig. 3.**
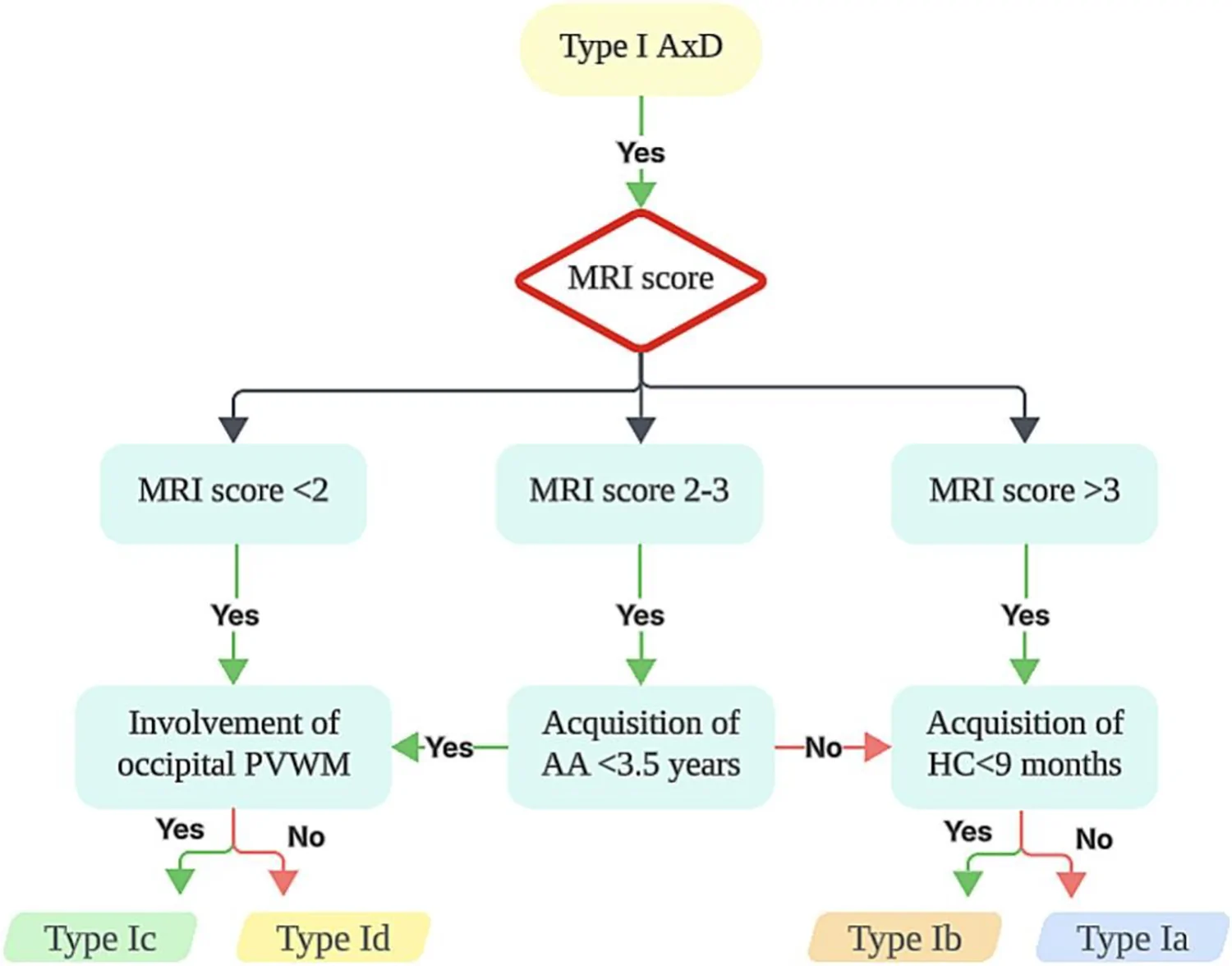
Prognostic flowchart in type I AxD.

**Table 1:** Involvement of brain MRI abnormalities across disease subtypes.

Variables	Ib *N*=12^a^	Ic *N*=7^a^	Ic/Id *N*=9^a^	Id N=9^a^	*p*-value^b^
Frontal periventricular WM	12 (100.0%)	7 (100.0%)	9 (100.0%)	7 (77.8%)	0.140
Frontal deep WM	12 (100.0%)	7 (100.0%)	9 (100.0%)	9 (100.0%)	/
Frontal subcortical WM	12 (100.0%)	5 (71.4%)	7 (77.8%)	2 (22.2%)	0.001
Parietal periventricular WM	11 (91.7%)	7 (100.0%)	7 (77.8%)	3 (33.3%)	0.005
Parietal deep WM	12 (100.0%)	5 (71.4%)	8 (88.9%)	2 (22.2%)	0.000
Parietal subcortical WM	7 (58.3%)	2 (28.6%)	0 (0.0%)	0 (0.0%)	0.002
Occipital periventricular WM	10 (83.3%)	6 (85.7%)	6 (66.7%)	1 (11.1%)	0.002
Occipital deep WM	11 (91.7%)	3 (42.9%)	5 (55.6%)	1 (11.1%)	0.002
Occipital subcortical WM	0 (0.0%)	0 (0.0%)	0 (0.0%)	0 (0.0%)	/
Temporal periventricular WM	10 (83.3%)	3 (42.9%)	5 (55.6%)	1 (11.1%)	0.010
Temporal deep WM	11 (91.7%)	4 (57.1%)	6 (66.7%)	1 (11.1%)	0.002
Temporal subcortical WM	4 (33.3%)	3 (42.9%)	0 (0.0%)	1 (11.1%)	0.106
Corpus callosum: Genu	7 (58.3%)	2 (28.6%)	4 (44.4%)	0 (0.0%)	0.029
Corpus callosum: Body	6 (50.0%)	2 (28.6%)	1 (11.1%)	0 (0.0%)	0.037
Corpus callosum: Splenium	1 (8.3%)	0 (0.0%)	0 (0.0%)	0 (0.0%)	1.000
PLIC	3 (25.0%)	2 (28.6%)	0 (0.0%)	0 (0.0%)	0.117
ALIC	6 (50.0%)	2 (28.6%)	5 (55.6%)	3 (33.3%)	0.675
Caudates	12 (100.0%)	6 (85.7%)	9 (100.0%)	9 (100.0%)	0.189
Putamina	12 (100.0%)	6 (85.7%)	9 (100.0%)	8 (88.9%)	0.329
Pallidi	11 (91.7%)	5 (71.4%)	7 (77.8%)	6 (66.7%)	0.557
Thalami	5 (41.7%)	3 (42.9%)	3 (33.3%)	2 (22.2%)	0.871
Cerebellum: Hilar region	10 (83.3%)	2 (28.6%)	3 (33.3%)	3 (33.3%)	0.036
Cerebellar WM	7 (58.3%)	3 (42.9%)	5 (55.6%)	2 (22.2%)	0.420
Mesencephalon	5 (41.7%)	2 (28.6%)	5 (55.6%)	2 (22.2%)	0.476
Pons	9 (75.0%)	2 (28.6%)	6 (66.7%)	5 (55.6%)	0.256
Medulla oblongata	12 (100.0%)	6 (85.7%)	9 (100.0%)	9 (100.0%)	0.189

Distribution of brain abnormalities seen as bilateral hyperintensity on T2WI images.

a *n* (%).

b Fisher’s exact test.

## Data Availability

The data that support the findings of this study are available on request from the corresponding author. The data are not publicly available due to privacy or ethical restrictions.

## Appendix A. Supplementary data

Supplementary data to this article can be found online at https://doi.org/10.1016/j.ymgme.2026.110189.

## References

[1] Brenner M, Johnson AB, Boespflug-Tanguy O, Rodriguez D, Goldman JE, Messing A, Mutations in GFAP, encoding glial fibrillary acidic protein, are associated with Alexander disease, Nat Genet. 27 (1) (Jan 2001) 117–120, 10.1038/83679. 11138011

[2] Prust M, Wang J, Morizono H, , GFAP mutations, age at onset, and clinical subtypes in Alexander disease, Neurology 77 (13) (Sep 27, 2011) 1287–1294, 10.1212/WNL.0b013e3182309f72. PMC3179649 21917775

[3] Russo LS, Aron A, Anderson PJ, Alexander’s disease: a report and reappraisal, Neurology 26 (7) (Jul 1976) 607–614, 10.1212/wnl.26.7.607. 180453

[4] Springer S, Erlewein R, Naegele T, , Alexander disease–classification revisited and isolation of a neonatal form, Neuropediatrics 31 (2) (Apr 2000) 86–92, 10.1055/s-2000-7479. 10832583

[5] Yoshida T, Nakagawa M, Clinical aspects and pathology of Alexander disease, and morphological and functional alteration of astrocytes induced by GFAP mutation, Neuropathology 32 (4) (Aug 2012) 440–446, 10.1111/j.1440-1789.2011.01268.x. 22118268

[6] Vaia Y, Mura E, Tonduti D, Type I Alexander disease: ipdate and validation of the clinical evolution-based classification, Mol Genet Metab. 138 (3) (Mar 2023) 107540, 10.1016/j.ymgme.2023.107540. 36804850

[7] Ashton NJ, Di Molfetta G, Tan K, Blennow K, Zetterberg H, Messing A, Plasma concentrations of glial fibrillary acidic protein, neurofilament light, and tau in Alexander disease, Neurol Sci. 45 (9) (Sep 2024) 4513–4518, 10.1007/s10072-024-07495-8. PMC11305938 38558318

[8] Jany PL, Agosta GE, Benko WS, , CSF and blood levels of GFAP in Alexander disease, eNeuro 2 (5) (Sep 2015), 10.1523/ENEURO.0080-15.2015. PMC4603256 26478912

[9] StatPearls. 2023.

[10] Hagemann TL, Powers B, Mazur C, , Antisense suppression of glial fibrillary acidic protein as a treatment for Alexander disease, Ann Neurol. 83 (1) (Jan 2018) 27–39, 10.1002/ana.25118. PMC5876100 29226998

[11] van der Knaap MS, Naidu S, Breiter SN, , Alexander disease: diagnosis with MR imaging, AJNR Am J Neuroradiol. 22 (3) (Mar 2001) 541–552. PMC7976831 11237983

[12] Harris PA, Taylor R, Thielke R, Payne J, Gonzalez N, Conde JG, Research electronic data capture (REDCap)–a metadata-driven methodology and workflow process for providing translational research informatics support, J Biomed Inform. 42 (2) (Apr 2009) 377–381, 10.1016/j.jbi.2008.08.010. PMC2700030 18929686

[13] Harris PA, Taylor R, Minor BL, , The REDCap consortium: building an international community of software platform partners, J Biomed Inform. 95 (Jul 2019) 103208, 10.1016/j.jbi.2019.103208. PMC7254481 31078660

[14] Mura E, Nicita F, Masnada S, , Alexander disease evolution over time: data from an Italian cohort of pediatric-onset patients, Mol Genet Metab. 134 (4) (Dec 2021) 353–358, 10.1016/j.ymgme.2021.11.009. 34865968

[15] Loes DJ, Hite S, Moser H, , Adrenoleukodystrophy: a scoring method for brain MR observations, AJNR Am J Neuroradiol. 15 (9) (Oct 1994) 1761–1766. PMC8333737 7847225

[16] Vrij-van den Bos S, Hol JA, La Piana R, , 4H leukodystrophy: a brain magnetic resonance imaging scoring system, Neuropediatrics 48 (3) (Jun 2017) 152–160, 10.1055/s-0037-1599141. 28561206

[17] Harting I, Garbade SF, Rosendaal SD, , Identification of PMD subgroups using a myelination score for PMD, Eur J Paediatr Neurol. 41 (Nov 2022) 71–79, 10.1016/j.ejpn.2022.10.003. PMC11348679 36368233

[18] Armangue T, Whitehead MT, Tonduti D, , Brainstem chipmunk sign: a diagnostic imaging clue across all subtypes of Alexander disease, AJNR Am J Neuroradiol 45 (6) (Jun 07, 2024) 769–772, 10.3174/ajnr.A8220. PMC11288593 38697787

[19] Kumar S, Sait H, Polipalli SK, Pradhan GS, Pruthi S, Kapoor S, Loes score: clinical and radiological profile of 22 patients of X-linked adrenoleukodystrophy: case series from a single center, Indian J Radiol Imaging. 31 (2) (Apr 2021) 383–390, 10.1055/s-0041-1734366. PMC8448211 34556923

[20] Tillema JM, Derks MG, Pouwels PJ, , Volumetric MRI data correlate to disease severity in metachromatic leukodystrophy, Ann Clin Transl Neurol. 2 (9) (Sep 2015) 932–940, 10.1002/acn3.232. PMC4574810 26401514

