# Supplementary material for: Neuroradiological patterns and prognostic implications in type I Alexander disease
Source: Mol Genet Metab. Author manuscript; Available in PMC 2026 Oct 13. (PMC13660844; doi:10.1016/j.ymgme.2026.110189)
Supplement: Supplementary file 1 [file NIHMS2215071-supplement-Supplementary_material_1__MRI_evaluation_protocol.pdf]

SUPPLEMENTARY MATERIAL

MRI

ID	______________________
Age at the time of MRI	______________________
Neuroradiology medical report	___________________________

	Normal	hyperintensity bilateral	hyperintensity monolateral	Cystic bilateral	Cystic monolat	Not performed
Frontal WM - Periventricular	☐	☐	☐	☐	☐	☐
Frontal WM - Deep	☐	☐	☐	☐	☐	☐
Frontal WM - Subcortical	☐	☐	☐	☐	☐	☐
Parietal WM - Periventricular	☐	☐	☐	☐	☐	☐
Parietal WM - Deep	☐	☐	☐	☐	☐	☐
Parietal WM - Subcortical	☐	☐	☐	☐	☐	☐
Occipital WM - Periventricular	☐	☐	☐	☐	☐	☐
Occipital WM - Deep	☐	☐	☐	☐	☐	☐
Occipital WM - Subcortical	☐	☐	☐	☐	☐	☐
Temporal WM - Periventricular	☐	☐	☐	☐	☐	☐
Temporal WM - Deep	☐	☐	☐	☐	☐	☐
Temporal WM - Subcortical	☐	☐	☐	☐	☐	☐
Corpus callosum - Genu	☐	☐	☐	☐	☐	☐
Corpus callosum - Body	☐	☐	☐	☐	☐	☐
Corpus callosum - Splenium	☐	☐	☐	☐	☐	☐
Projection fibers - PLIC	☐	☐	☐	☐	☐	☐
Projection fibers - ALIC	☐	☐	☐	☐	☐	☐
Basal ganglia - Caudates	☐	☐	☐	☐	☐	☐
Basal ganglia - Putamina	☐	☐	☐	☐	☐	☐
Basal ganglia - Pallidi	☐	☐	☐	☐	☐	☐
Thalami	☐	☐	☐	☐	☐	☐
Cerebellum - Hilar region	☐	☐	☐	☐	☐	☐
Cerebellum - Cerebellar WM	☐	☐	☐	☐	☐	☐
Brainstem - Mesencephalon	☐	☐	☐	☐	☐	☐
Brainstem - Pons	☐	☐	☐	☐	☐	☐
Brainstem - Medulla Oblungata	☐	☐	☐	☐	☐	☐
Spinal Cord	☐	☐	☐	☐	☐	☐

"Mass lesions" (nodular asymmetric highly hyperintense lesions)

	Absent	Present	Monolateral	Bilateral
Brainstem - Mesencephalon	☐	☐	☐	☐
Brainstem - Pons	☐	☐	☐	☐
Brainstem - Medulla Oblungata	☐	☐	☐	☐
Other - Please specify	☐	☐	☐	☐

____

Enhancement

◯ Present
◯ Absent
◯ Not performed

	Front-periv	Front-deep	Front-subc	Pariet -periv	Pariet -deep	Pariet -subc	Occip -periv	Occip -deep	Occip -subc	Temp -periv	Temp -deep	Temp -subc
Present	☐	☐	☐	☐	☐	☐	☐	☐	☐	☐	☐	☐

	CC-genu	CC-body	CC-splenium	PLIC	ALIC	Caud	Putam	Pall	Thal
	☐	☐	☐	☐	☐	☐	☐	☐	☐

	Hili	Dentates	Cereb WM	Mesen	Pons	Medul Obl	Spin Cord
	☐	☐	☐	☐	☐	☐	☐

Diffusion

◯ Normal
◯ Abnormal
◯ Not performed

	Front-periv	Front-deep	Front-subc	Pariet -periv	Pariet -deep	Pariet -subc	Occip -periv	Occip -deep	Occip -subc	Temp -periv	Temp -deep	Temp -subc
Increased	☐	☐	☐	☐	☐	☐	☐	☐	☐	☐	☐	☐

	CC-genu	CC-body	CC-splenium	PLIC	ALIC	Caud	Putam	Pall	Thal
	☐	☐	☐	☐	☐	☐	☐	☐	☐

	Hili	Dentates	Cereb WM	Mesen	Pons	Medul Obl	Spin Cord
	☐	☐	☐	☐	☐	☐	☐

	Front-periv	Front-deep	Front-subc	Pariet -periv	Pariet -deep	Pariet -subc	Occip -periv	Occip -deep	Occip -subc	Temp -periv	Temp -deep	Temp -subc
Reduced	☐	☐	☐	☐	☐	☐	☐	☐	☐	☐	☐	☐

	CC-genu	CC-body	CC-splenium	PLIC	ALIC	Caud	Putam	Pall	Thal
	☐	☐	☐	☐	☐	☐	☐	☐	☐

	Hili	Dentates	Cereb WM	Mesen	Pons	Medul Obl	Spin Cord
	☐	☐	☐	☐	☐	☐	☐

Atrophy Score	0	1	2
Supratentorial (bicaudate ratio) (tab below)	○	○	○
Corpus callosum*	○	○	○
Brainstem (pons diameter) (tab below)	○	○	○
Cerebellum**	○	○	○
Spinal cord	○	○	○

PONS DIAMETER SCORE	2-3y	4-5y	6-7y	8-9y	10-12y	13-14y	15-19y	>23y
0	>18	>19	>19	>20	>20	>21	>21	>22
1	17,99-16,01	18.99-16.01	18.99-17.01	19.99-17.01	19.99-18.01	20.99-18.01	20.99-19.01	21.99-20.01
2	<16	<16	<17	<17	<18	<18	<19	<20

SUPRATENTORIAL ATROPHY SCORE BASED ON BICAUDATE RATIO				
	2-9Y	10-18Y	19-23Y	≥24Y
0	<0.085	<0.090	<0.095	<0.100
1	0.085–0.100	0.090–0.105	0.095–0.110	0.100–0.115
2	>0.100	>0.105	>0.110	>0.115

*Corpus callosum atrophy : 0 = normal, 1 = slightly thinned, 2 = severely thinned

**Cerebellar vermis and cerebellar hemispheres: 0 = normal, 1 = mild atrophy with slightly enlarged interfolial spaces, 2 = severe atrophy with clearly enlarged interfolial spaces

Bicaudate ratio: the ratio of width of two lateral ventricles at the level of the head of the caudate nucleus to distance between outer tables of skull at the same level

TOTAL ATROPHY SCORE ______________________________
