# Supplementary material for: Neuroradiological patterns and prognostic implications in type I Alexander disease
Source: Mol Genet Metab. Author manuscript; Available in PMC 2026 Oct 13. (PMC13660844; doi:10.1016/j.ymgme.2026.110189)
Supplement: Supplementary file 2 [file NIHMS2215071-supplement-Supplementary_material_2__MRI_evaluation_protocol.docx]

SUPPLEMENTARY MATERIAL

METHODS

WM abnormalities were assessed on T2-weighted images, with FLAIR sequences used to support the evaluation. Hemispheric white matter involvement was defined as "bilateral T2-hyperintensity" when more than 50% of the WM in the specific area was involved, bilaterally. T1-weighted images with gadolinium injection were used to assess the presence of pathological contrast enhancement (CE), and diffusion sequences were evaluated to determine the presence of either increased or restricted diffusion.

Atrophy was assessed at the supratentorial level (using the bicaudate ratio), as well as in the corpus callosum, brainstem, cerebellum, and, when available, the spinal cord, following the approach of peer-reviewed atrophy scoring systems used in other leukodystrophies.[14, 15] An atrophy score was calculated, excluding the spinal cord, due to the frequent absence of spinal cord MRI sequences.

**Table 1.**

| **Patient** | **Type of AxD** | **Age at onset** | **Age at MRI** | **MRI score** |
| --- | --- | --- | --- | --- |
|  |  |  |  |  |
| P1 | Ib | 1.50 | 3.00 | 4 |
| P2 | Ib | 1.00 | 3.50 | 2 |
| P3 | Ib | 0.10 | 1.70 | 5 |
| P4 | Ib | 0.40 | 0.70 | 5 |
| P5 | Ib | 1.16 | 1.90 | 5 |
| P6 | Ib | 1.50 | 1.75 | 5 |
| P7 | Ib | 0.50 | 3.90 | 5 |
| P8 | Ib | 0.17 | 1.20 | 2 |
| P9 | Ib | 0.75 | 2.90 | 5 |
| P10 | Ib | 0.10 | 2.10 | 3 |
| P11 | Ib | 1.40 | 4.20 | 5 |
| P12 | Ib | 0.50 | 1.50 | 3 |
| P13 | Ic | 0.33 | 2.80 | 2 |
| P14 | Ic | 0.80 | 1.70 | 1 |
| P15 | Ic | 1.50 | 4.90 | 5 |
| P16 | Ic | 1.50 | 2.20 | 5 |
| P17 | Ic | 7.00 | 9.20 | 0 |
| P18 | Ic | 0.75 | 2.60 | 0 |
| P19 | Ic | 0.33 | 2.30 | 1 |
| P20 | Ic/Id | 1.25 | 3.60 | 3 |
| P21 | Ic/Id | 1.50 | 5.10 | 3 |
| P22 | Ic/Id | 1.50 | 4.70 | 3 |
| P23 | Ic/Id | 0.70 | 4.00 | 3 |
| P24 | Ic/Id | 1.10 | 2.30 | 1 |
| P25 | Ic/Id | 0.75 | 1.80 | 1 |
| P26 | Ic/Id | 1.83 | 1.83 | 3 |
| P27 | Ic/Id | 2.50 | 6.30 | 0 |
| P28 | Ic/Id | 1.75 | 2.30 | 2 |
| P29 | Id | 1.50 | 2.30 | 0 |
| P30 | Id | 1.70 | 5.40 | 1 |
| P31 | Id | 6.17 | 7.00 | 1 |
| P32 | Id | 0.40 | 0.40 | 0 |
| P33 | Id | 7.00 | 7.80 | 1 |
| P34 | Id | 1.50 | 4.10 | 0 |
| P35 | Id | 2.00 | 3.90 | 1 |
| P36 | Id | 1.20 | 2.20 | 0 |
| P37 | Id | 0.33 | 1.80 | 2 |
